# Follicular dendritic cell sarcoma: a report of six cases and a review of the Chinese literature

**DOI:** 10.1186/1746-1596-5-67

**Published:** 2010-10-11

**Authors:** Haiwei Wang, Zhansan Su, Zhongliang Hu, Jifang Wen, Baoan Liu

**Affiliations:** 1 Department of Pathology, Xiangya Basic Medical School, Central South University, Changsha, Hunan Province, 410013, China; 2 Department of Pathology, Xiangya Third Hospital, Central South University, Changsha, Hunan Province, 410013, China

## Abstract

**Goals:**

The main purpose of this study is to broaden the clinicopathological spectrum and increase recognition of follicular dendritic cell sarcoma (FDCS) through analysis of the clinical and pathological features of 50 cases.

**Methods:**

The clinicopathological features of total 50 cases of FDCS were analyzed including a review of 44 cases reported in Chinese literature before October 2009 and six original cases from the pathology files conducted by the authors.

**Results:**

The youngest patient came under observation in this study is only seven years old. Including the cases contributed by the authors, our literary review indicated that male dominated the tumor cases (M: F = 3: 2). 28 cases (56%) present with this disease in extranodal sites. Tumor cells demonstrated positive staining for the follicular dendritic cell markers CD21 (47/49), CD35 (43/45), CD23 (20/23) and CD68 (23/25). In situ hybridization for Epstein-Barr virus-encoded RNA was performed in 10 cases. Nevertheless, EBV expression was absent in all these cases. The follow-up analysis of all cases shows that 26 (81.2%) patients were alive and disease free; 6 (18.8%) patients were alive with recurrent disease or metastasis; and nobody had died of this disease at the time of last follow-up.

**Conclusions:**

The diagnosis of the FDCS is based on the findings of morphology and immunohistochemistry. The FDCS occurred in China should be viewed and treated as a low-grade sarcoma, and the role of the EBV in the pathogenesis of this tumor is still uncertain. There is a possibility that the tumor might be racial or geographic correlated, because most cases were reported from Eastern Asia area; it's particular the case of the liver or spleen tumor.

## Introduction

Classified as accessory cells of the immune system, the follicular dendritic cells (FDC), also known as dendritic reticulum cells, are essential for the function of antigen presentation and germinal center reaction regulation. Follicular dendritic cell sarcoma (FDCS) is an uncommon tumor that usually arises in lymph nodes, especially in the cervical, mediastinal, and axillary areas, but also in extranodal sites. The existence of a primary neoplasm of the follicular dendritic cell was first recognized in 1986 by Monda et al [1]; since then, scattered cases have been reported in literatures. Approximately 150 cases have been reported in English literature so far. The scarcity may be partially due to under-recognition of this entity. In recent years there has been an increasing interest in this specific type of neoplasm due to the emerging availability of specific antibodies that can confirm the FDC lineage.

To add to the existing literature on FDCS to deepen the understanding of the disease, we analyzed the clinical characteristics, pathologic features, immunophenotypic profile, treatments and outcome of 50 cases including our 6 cases and 44 cases from reviewing the Chinese literatures on the FDCS.

## Materials and methods

The present study included a total of 50 patients with FDCS. Having not been reported previously, the first 6 cases were retrieved from the pathological files of the Department of Pathology, Xiangya Hospital of Central South University between 2004 and 2009. The other cases were obtained through the major indexed literature database CNKI (China National Knowledge Infrastructure) between 1994 and 2009 by searching the key words like dendritic cell, follicular dendritic cell, and sarcoma. The clinical characteristics of all cases of FDCS were summarized in additional file 1.

Routine histopathological and immunohistochemical analyses were performed from formalin-fixed, paraffin-embedded specimens. The panel of antibodies mainly included CD21, CD35, CD23, CD68, CD20, EMA, Vimentin, S-100 protein, Clusterin, and LMP-1. All the antibodies were products of DAKO and staining was performed with the DAKO En Vision Kit. The sections were developed with 3,3'-diaminobenzidine tetrahydrochloride and counterstained with hematoxylin.

In situ hybridization for Epstein-Barr virus (EBV) -encoded RNA (EBER) was performed on paraffin sections using an EBER in situ hybridization and detection kit (DAKO), and the staining procedure was precisely done as recommended by the manufacturer. EBV-associated nasopharyngeal carcinoma was served as positive control.

## Results

### Clinical features

The present study contains 30 males and 20 females. The mean age of the patients was 44 years, ranging from 7 to 75. The 7 years old patient was the youngest observed so far. The constitutional symptoms, including low-grade fever, weight loss, epigastralgia, abdominal fullness, slight anemia, and so on, were nonspecific in patients with FDCS. Patients often presented with a slow-growing, well-circumscribed, painless mass. One case was complicated by Castleman's disease, which was discovered in chest wall. Of all cases summarized in this review, the most common site of tumor involvement was lymph node, for 22 cases (44%). The others (28, 56%) were located in extranodal sites, including liver (5), gastrointestinal tract (5), nasal pharynx (5), spleen (4), parotid gland (2), greater omentum (2), cavitas pelvis and abdominal cavity (2), tonsil (1), adrenal gland (1), and chest wall (1).

### pathologic features

#### Morphological findings

**Figure 1:**
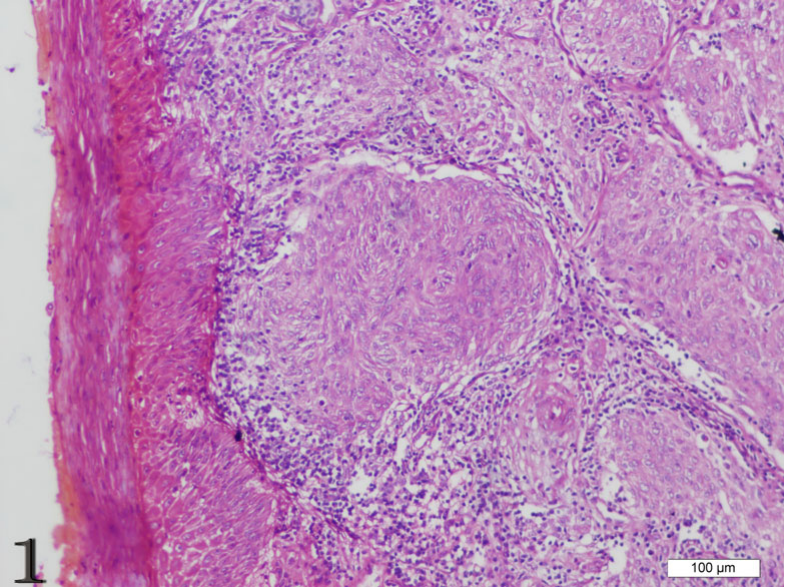
**Case 1 FDCS of the tonsil, the spindle cells are arranged in a storiform and whorled patterns**. (H&E stain).

**Figure 2:**
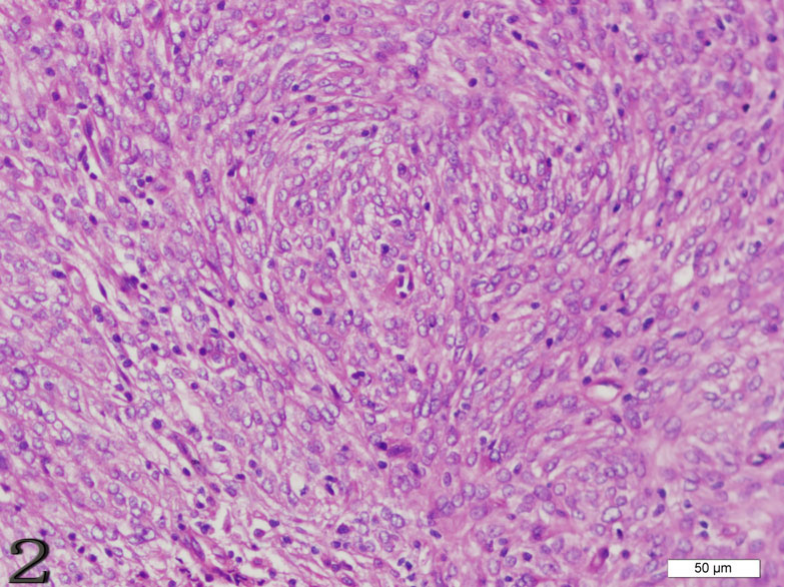
**Case 1 FDCS of the tonsil, individual cells possess characteristic features, including a delicate nuclear membrane, vesicular nuclei, small but distinct nucleoli and indistinct cell borders**. (H&E stain).

**Figure 3:**
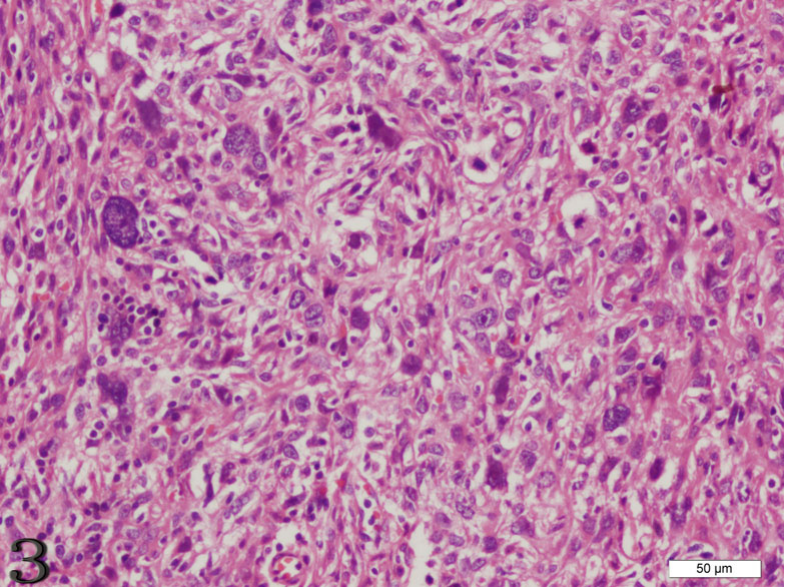
**Case 3 FDCS of the liver, the giant cells and mitotic figures are identified occasionally**. (H&E stain).

The gross measurements of this tumor varied from case to case, ranging from 0.6 to 21 cm. Macroscopically, the tumor was characteristically well circumscribed or even had a capsule. It was soft to slightly firm and might contain areas of necrosis and hemorrhage. Microscopically, the tumor was composed of oval to spindle cells, regardless of the sites of involvement, arranging in storiform, whorled, and fasciclar patterns (Figure 1). Individual cells possessed characteristic features, including slightly eosinophilic, fibrillary cytoplasm, a delicate nuclear membrane, vesicular nuclei, small but distinct nucleoli and indistinct cell borders with occasionally multinucleated forms (Figure 2). A common and highly characteristic feature was the sprinkling across the entire tumor of small lymphocytes, occasionally leading to a misdiagnosis of lymphoma. The lymphocytes sometimes also showed cuffing around the blood vessels. The giant cells and mitotic figures were identified occasionally (Figure 3). Irregular hemorrhage and coagulative necrosis were observed in some cases.

**Figure 4:**
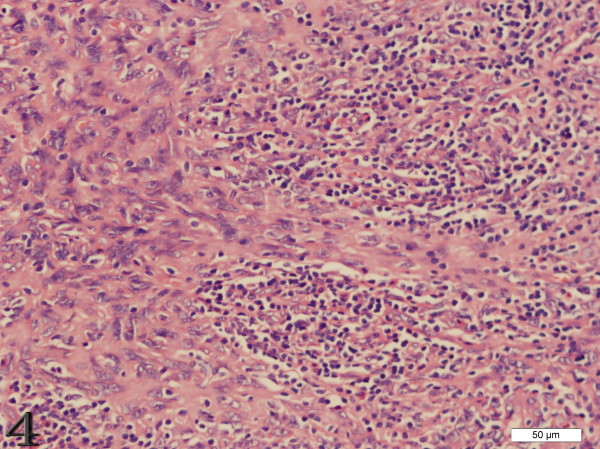
**Case 4 FDCS of the lymph node, the spindle cells are dispersed in distribution, scattered by a prominent lymphoplasmacytic infiltrate (H&E stain)**.

Three out of nine cases that involves the liver and spleen were distinct in that they often had histological features resembling inflammatory pseudotumor[2-4]. In these cases, the spindle cells were dispersed in distribution, scattered by a prominent lymphoplasmacytic infiltrate(Figure 4).

#### Immunophenotypic findings

**Figure 5:**
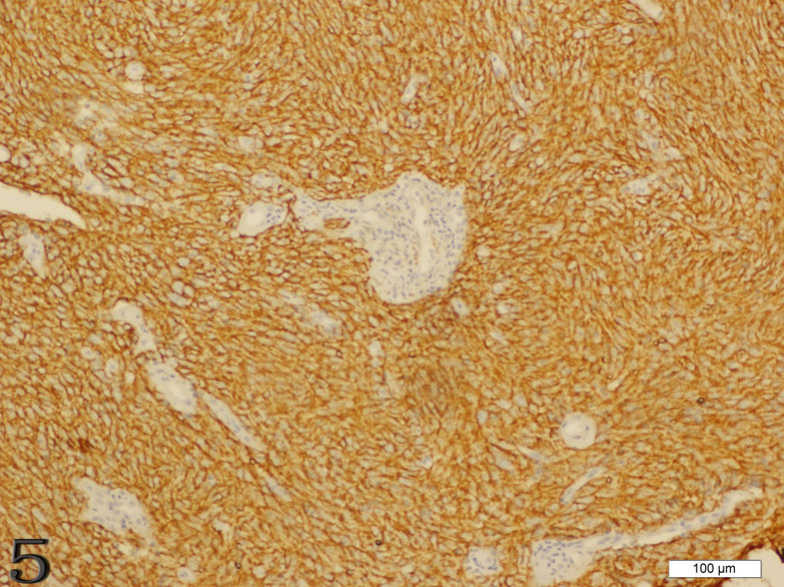
**Case 1 FDCS of the tonsil, the tumor cells are immunoreactive with CD21 strongly and diffusely**.

**Figure 6:**
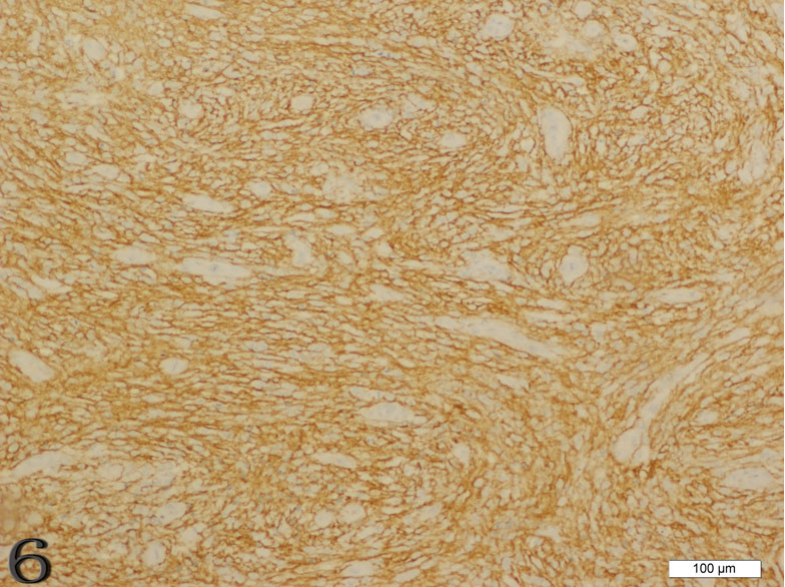
**Case 4 FDCS of lymph node, the tumor cells were positive for CD35 strongly and diffusely**.

Immunohistochemically, the FDCS exhibited unique histological immunophenotypic features (Figures 5 and 6). Tumor cells demonstrated positive staining for the follicular dendritic cell markers CD21 (49/49), CD35 (43/45), CD23 (20/23), CD68 (23/25), Vimentin (22/28), and 18 cases showed immunostaining for S-100 protein. Positive staining for EMA was observed in 16 cases. EBV-latent membrane protein (LMP-1) only showed positive staining in 1 of 7 cases[5]. Other positive but uncommon immunohistochemical markers included HLA-DR, SMA, Clusterin, NSE and LCA.

#### Ultrastructural findings

Electron microscopic examination was available in 4 cases in published literature [6-8]. Tumor cells, admixed with numerous inflammatory cells, demonstrated elongated nuclei and often with finely dispersed chromatin. Scattered lysosomes and well-developed Glogi were noted in some cells. The most distinctive feature, however, was numerous long, slender cytoplasmic processes joined by mature desmosomes.

#### EBER in situ hybridization

In situ hybridization for Epstein-Barr virus (EBV)-encoded RNA was performed in 10 cases. Nevertheless, EBV expression was absent in all these cases[2,9,10].

### Treatment and outcome

Although various treatments including surgery, chemotherapy, radiation therapy, and combinations of these modalities have been used, complete surgical resection is still the mainstay of treatment for primary FDCS for most patients. In all 50 cases studied in this paper, the patients were all treated by surgical excision, while adjuvant radiotherapy or chemotherapy was supplemented in 13 cases. Follow-up information was available in 32 patients, with a time interval ranging from 1 month to 5 years. Twenty-six (81.2%) patients were alive and disease free after the treatment, 6 (18.8%) patients were alive with recurrent disease or metastasis, and nobody had died of this disease at the time of the last follow-up.

## Discussion

FDCS is an extremely rare tumor and has been recognized with increasing frequency. Most of the data on FDCS are based on case reports or small case series since the first report in 1986. In recent years, FDCS is receiving increasing attention because of the availability of more sensitive markers to confirm the FDC lineage. Interestingly, there is a possibility that the tumor has a race-or geographic-correlation, because 17 cases were from Japan among the worldwide 50 cases reviewed in our study, despite the fact that accurate information regarding ethnic and living environment of the patients were not available[11]. The tumor was thought to mainly affect lymph nodes, with cervical and axillary regions being the most common sites. However, more than half of the reported cases in our review have been located in extranodal sites, including palate, pharynx, tonsil, thyroid, mediastinum, spleen, gastrointestinal tract, liver[2-10,12-32]. Therefore, FDCS presenting in extranodal sites are worthy of more attention than ever.

At present, there is no definite etiology for most cases of FDCS. Cytogenetic abnormalities have been described in one case of FDCS in the spleen[33], displaying multiple clonal unbalanced chromosomal translocations, including loss of Xp. It was proposed this finding might be early evidence of the role of Xp in the development of this tumor. Interestingly, Castleman^'^s disease has been found in association with FDCS for a minority of case[26,34], which suggests that it may represent a precursor lesion. FDC proliferation and dysplastic changes occurring in Castleman^'^s disease can form the background from which a FDCS develops. Perhaps the tumor arises through the sequence of hyperplasia-dysplasia-neoplasia. However, with only one patient presented FDCS complicated by Castleman's disease in our study[26], additional studies will be required to decide whether there is a relationship between Castleman's disease and FDCS. A possible role for p53 in the transformation process has also been proposed [35], with overexpression of p53 protein noted in FDCS as well as an increased number of weakly p53-positive spindle cells in a hyaline-vascular Castleman^'^s disease specimen.

EBV has been documented to be positive in a small percentage of FDCS cases[36]. It is no wonder that FDCS shows association with this virus because FDCS expresses CD21 (EBV receptor) and can be experimentally infected by EBV. In the 6 cases conducted by our own team, however, we didn't detect evidence of the virus by in situ hybridization for EBER-1 gene. Although the association of FDCs tumor with EBV seems to vary in different organs, FDCS of liver seems to be strongly presented in a peculiar way. These differentiated features have also been noted in splenic follicular dendritic cell tumors [37]. Thus, the role of EBV remains unclear in the pathogenesis of FDCS tumors. Longer follow-up of more patients is necessary to better define the characteristics of this peculiar tumor. Latent membrane protein-1(LMP-1) is an integral membrane protein, and is encoded by the LMP-1 gene of EBV. LMP-1 is considered to be a viral oncogene because of its capacity to transform rodent fibroblasts in vitro and render them tumorigenic in nude mice[38]. However, only one case in our literature review shows positive staining for LMP-1. Therefore, the clinicopathological significance of the LMP-1 gene in FDCS tumors warrants further investigation due to the fact that only a small amount of clinicopathological data has been documented so far.

The pathologic characteristics and immunophenotypic profile of FDCS in our series are similar to those in the literature. The most common histological feature is the presence of oval to spindle cells with elongated nuclei, delicate, dispersed chromatin and pale eosinophilic cytoplasm. Lymphocytes may gather around blood vessels, creating a cuffing pattern. Concentric whorl is also a characteristic growth pattern. Scattered multinucleated tumor cells may also be present. All these various morphologic features must be considered in the differentiated diagnosis of FDCS.

To Sum up the literature, CD21 and CD35 are the most widely used markers. Other useful markers are vimentin, CD23, CD68, S-100 protein, fascin, Ki-M4p and Ki-FDC1p; however, these are unspecific. FDCS typically lacks expression of CD1a, desmin and CD45, which allows their differential diagnosis with interdigitating dendritic cell tumors, Langherhans cell tumors, histiocytic and lymphoid neoplasias. So the expression of non-typical FDCS markers should be taken into account in the differential diagnosis with other neoplasms.

The diagnosis of FDC tumor is established basing on the findings of morphology and immunohistochemistry. Ultrastructural studies may be helpful but are not indispensable for accurate diagnosis. All neoplasms in the differential diagnosis lack follicular dendritic cell differentiation and are easily excluded if FDCS is considered and immunohistochemical staining with FDC markers is applied.

The optimal treatment for FDCS is yet to be found due to the limited experience. Current best-available approach is to apply therapeutic guidelines similar to those used for soft tissue sarcomas of high grade. Our study does show some trends though. Both the analytic literature review and the cases conducted by ourselves suggest that FDCS can be effectively treated by surgery and no radiotherapy or chemotherapy after radical excision is required.

Lymph nodes, lung, and liver are the most common sites for metastasis. In case studies conducted by our own team members, the clinical course of follicular dendritic cell sarcomas is akin to that of low-grade tumors. These figures are probably underestimates, because many cases in the literature had short follow-up periods.

To conclude, FDCS is an extremely rare tumor of lymph nodes and extranodal tissues. Because of the scarcity of the identified cases, it is probably under recognized, particularly when happened in extranodal sites. Recently, the diagnosis accuracy has been significantly improved, thanks to the aid of IHC analysis and the two most reliable FDC markers CD21 of and CD35. Once FDCS is suspected histologically, immunohistochemical stains for follicular dendritic cell differentiation should be performed to avoid the risk of misdiagnosis. As noted previously, FDCS occurring in the liver or spleen may represent a variant subset that differs from the FDCS in other sites, producing a histological picture similar to inflammatory pseudotumor(IPT). They probably have different characteristics and causes. So it is important to consider the possibility of an IPT-like FDC tumor when making a diagnosis of a hepatic/splenic IPT-like lesion.

FDCS has a slight recurrent and metastatic potential and it should be viewed as a low-grade malignancy. Literature shows that therapeutic modalities in this disease varied widely although complete surgical resection was often included. In addition, adjuvant chemotherapy and radiotherapy may be utilized; but the value of these adjuvant treatments to effectively improve survival rates has not been convincingly demonstrated.

Most of the data on FDCS are based on single case report or small-group case series, further investigations are required to address the following questions: (1) more systematic data are needed to understand the role of the EBV in the pathogenesis of this tumor. (2) Further detailed cytogenetic studies of FDCS might reveal a specific chromosomal profile of these tumors, which in turn will yield new insights into the biology of FDCS and provide the pathologist with independent genetic markers. (3) additional studies needed to decide whether Asians would be susceptible to this disease.

## Competing interests

The authors declare that they have no competing interests.

## Authors' contributions

WHW carried out the in situ hybridization, performed the literature review, acquired photomicrographs, and drafted the manuscript. LBA conceived and designed the study. LBA and SZS conducted and reviewed the final histopathological diagnosis. HZL carried out and evaluated the immunohistochemical stains. WJF revised the manuscript for important intellectual content. All authors read and approved the final manuscript.

## Supplementary Material

Additional file 1 **The clinical characteristics of 50 cases of FDCS**. The clinical characteristics of all cases of FDCS were summarized in additional file 1. Click here for file
