# Supplementary material for: Follicular dendritic cell sarcoma: a report of six cases and a review of the Chinese literature
Source: Diagn Pathol. 2010 Oct 11;5:67. doi: 10.1186/1746-1596-5-67 (PMC2964601; doi:10.1186/1746-1596-5-67)
Supplement: Additional file 1 — The clinical characteristics of 50 cases of FDCS. The clinical characteristics of all cases of FDCS were summarized in additional file 1. [file 1746-1596-5-67-S1.DOC]

Table 1. The clinical characteristics of 50 cases of FDCS

| case | Sex/age(y) | clinical present | site | size(cm) | treatment | outcome |
| --- | --- | --- | --- | --- | --- | --- |
| 1 | F/47 | Pharyngeal paraesthesia | Tonsil | 2.0×2.0×1.8 | Excision | Alive and disease free at 15 months |
| 2 | M/73 | Mass in left upper quadrant noted by health examination | Spleen | 7cm in diameter | Excision | Alive and disease free at 38 months |
| 3 | M/41 | Vague pain of right lower quadrant | Liver | 13×10×9 | Excision | Alive and disease free at 10 months |
| 4 | F/28 | Enlarging mass in left axilla | Lymph node | 5×5×3 | Excision | Alive and disease free at 56 months |
| 5 | M/50 | Epigastric pain | Spleen | 13×8×6 | Excision | Alive and disease free at 26 months |
| 6 | M/24 | Mass in spleen noted by health examination | Spleen | 6.5×6.0×5.0 | Excision | Metastasis to the skin 24 months after the primary tumor removed |
| 7 | M/19 | Nasal bleeding | Nasal cavity | Peanut | Excision,followed by cryosurgery | Alive and disease free at 11 months |
| 8 | M/37 | Pharyngeal paraesthesia | Right tonsil | 1.5×1.5×1.5 | Right tonsillectomy,  followed by chemotherapy | Alive and disease free at 36 months |
| 9 | F/25 | Tetter, oliguria, generaldropsy | Mesentery | 5.4×5×4.8,  6×3.5×3.2,  1.2×1.2×1.1 | Excision followed radiotherapy | NA |
| 10 | M/42 | Painaroundthenavel | Sinus ventriculi | 12×7×6 | Excision | NA |
| 11 | M/63 | Painless mass of left upper quadrant | Mesojejunum | 15cm in diameter | Excision | NA |
| 12 | M/43 | Intermittent pain of right lower quadrant | Ileocecal junction | 4.0×4.0×3.5 | Excision | NA |
| 13 | F/28 | Upper abdominal pain | Cardia | 15×15 | Excision followed chemotherapy | Recurrence at 3 mos in the left lobe of liver, alive and disease free at 5 years |
| 14 | M/42 | Abdominal pain | Greater omentum | 12×7 | Excision | Alive and disease free at 2 years |
| 15 | M/75 | Abdominal pain in right upper quadrant | Liver | 4.0×3.0×2.5 | Excision | Alive and disease free at 2 years |
| 16 | F/57 | Abdominal pain in right upper quadrant | Liver | 11×10×10 | Excision | NA |
| 17 | M/33 | Upper abdominal pain | Liver | 13×15 | Excision | NA |
| 18 | F/36 | Swelling of left neck | Left neck | 6×6×4 | Excision | Local recurrence thrice |
| 19 | F/73 | Diffuse lymph node enlargement | Diffuse | 2.0×1.5×1.0 | NA | Alive and disease free at 6 moths |
| 20 | M/53 | Swelling of right neck | Right neck | 3.0×2.5×1.0 | NA | NA |
| 21 | F/7 | Swelling of right neck | Underneath the right auricular lobule | 2.8×1.6 | Excision | NA |
| 22 | F/39 | Swelling of left neck | Left neck | 4.8×3.0×2.4 | Excision followed chemotherapy (CHOP) | Alive and disease free at 11 moths |
| 23 | M/60 | Pharyngeal paraesthesia and headache of intermittence | Pharyngeal portion | 2.0×1.0×0.4 | Excision followed radiotherapy | Alive and disease free at 2 years |
| 24 | F/35 | Swelling of right pharyngeal portion and accompanying dysphagia | Right pharyngeal portion | 5.0×4.5×3.0 | Excision followed chemotherapy and radiotherapy | Alive and disease free at 1 years |
| 25 | M/19 | Swelling of right neck | Right neck | Biopsy | Excision followed chemotherapy | Local recurrence at 1 months |
| 26 | F/45 | Painless mass of right axilla | Right axillary fossa | Biopsy | Excision followed chemotherapy（CHOP） | NA |
| 27 | M/59 | Swelling of right neck | Right neck | 6×5 | Excision followed chemotherapy(NPP) | Alive and disease free at 7 months |
| 28 | M/24 | Mass in the neck and rachialgia | Neck | 4×4 | Excision followed chemotherapy(EP and CHOP) | Multiple metastases to bones |
| 29 | M/16 | Erosion in oral cavity and pudendum | Right adrenal gland | 5cm in diameter | Excision | NA |
| 30 | F/41 | Abdominal distension and anepithymia | Colon | 6×6×5 | Excision | NA |
| 31 | F/46 | General fatigue  and abdominal distension | Stomach  mesostenium  right uterine adnexa | 21×12×10,  14×12×9.5,  6×4.5×3.5 | Excision | Alive and disease free at 6 months |
| 32 | M/55 | Mass in right parotid gland | Right parotid gland | 5cm in diameter | Excision | NA |
| 33 | M/63 | Swelling of abdomen | Mesojejunum | 15×8×7 | Excision | NA |
| 34 | M/60 | Swelling of left upper quadrant  of abdomen | Mesostenium | 7×6×3 | Excision | NA |
| 35 | M/64 | Enlarging mass in chest wall | Chest wall | 16×13×10 | Excision | Alive and disease free at 15 months |
| 36 | F/72 | Enlarging mass in inguinal groove | Left inguinal groove | 7×5×5 | Excision | NA |
| 37 | M/42 | Painless mass of left neck | Left neck | 7.0×4.5×1.0 | Excision(first) and excision followed radiotherapy  (second) | Local recurrence twice in 5 years |
| 38 | F/36 | Mass in left mandible | Left mandible | 3.3×2.6 | Excision | Alive and disease free at 5 months |
| 39 | F/32 | Mass in left neck | Left parotid | 4.5×3.5 | Excision followed chemotherapy (CHOP) | Alive and disease free at 20 months |
| 40 | M/37 | Painless mass of left neck | Left neck | 6.0×4.5 | Excision | Alive and disease free at 41 months |
| 41 | M/34 | Mass in left neck | Left neck | 5.5×4.0 | Excision | Alive and disease free at 52 months |
| 42 | M/40 | Space-occupying mass of right liver noted by health examination | Right liver | 5.0×4.5×4.0 | Excision | Alive and disease free at 3 months |
| 43 | F/35 | Mass in abdomen noted by accident | Abdomen | 10.5×5.5×4.0 | Excision | Alive and disease free at 24 months |
| 44 | M/48 | Mass in right mandible | Right mandible | 7×4 | Excision followed chemotherapy(CHOP) | Alive and disease free at 12 months |
| 45 | M/68 | Fervescence, chest distress, splenohepatomegalia | Inguinal groove | * | Excision | NA |
| 46 | M/55 | Diffuse lymph node enlargement | Axillary cavity | * | Excision | Alive and disease free at 4 months |
| 47 | F/62 | Recurrent fever | Axillary cavity | * | Excision | NA |
| 48 | F/71 | Diffuse lymph node enlargement and pruritus | Axillary cavity | * | Excision | Alive and disease free at 1 months |
| 49 | M/36 | Pharyngeal malaise | Nasal pharynx | * | Excision | Alive and disease free at 5 months |
| 50 | F/32 | Epigastric pain and hypodynamia | Spleen | * | Excision | NA |

NA:not available;*the diameter of the tumor ranges from 0.6 to 5.0cm.
